# Autologous SVF therapy modulates neuroinflammation in ALS: phase I trial demonstrating safety and CSF biomarker dynamics

**DOI:** 10.3389/fnagi.2026.1784115

**Published:** 2026-03-31

**Authors:** Ruixia Li, Lin Wang, Wendi Bu, Xu Zhang, Xiaojing Li, Jing Li, Jiahui Shen, Jingwei Li, Zenglin Cai

**Affiliations:** 1 Department of Neurology, Suzhou Hospital, Affiliated Hospital of Medical School, Nanjing University, Suzhou, China; 2 Suzhou Research Center of Medical School, Suzhou Hospital, Affiliated Hospital of Medical School, Nanjing University, Suzhou, China; 3 Department of Neurology, Gulou School of Clinical Medicine, Nanjing Medical University, Nanjing, Jiangsu, China; 4 Department of Neurology, Suqian Zhongwu Hospital, Suqian, China; 5 Department of General Surgery, Suzhou Hospital, Affiliated Hospital of Medical School, Nanjing University, Suzhou, China

**Keywords:** amyotrophic lateral sclerosis, amyotrophic lateral sclerosis functional rating scale, glial fibrillary acidic protein, neurofilament light chain, stromal vascular fraction

## Abstract

**Background:**

Amyotrophic lateral sclerosis (ALS) is a neurodegenerative disorder with limited treatments. Stromal vascular fraction (SVF), a cell population derived from autologous adipose tissue, exhibits multimodal immunomodulatory and neuroprotective properties, positioning it as a promising therapeutic candidate.

**Methods:**

This trial aimed to assess autologous stromal vascular fraction (SVF) safety and efficacy in patients with ALS. 26 patients received combined intravenous (0.5 × 10*6* cells/kg) and intrathecal (20 × 10^6^ cells) autologous SVF (An exploratory second dose of SVF was administered intrathecally to three patients 45 days later). The trial is registered with the Chinese Clinical Trial Registry (ChiCTR2400091754).

**Results:**

SVF administration was well-tolerated. Five mild adverse events (adverse events, AEs) (subcutaneous bleeding, headache, and low-grade fever) occurred, with no serious AEs reported. Although ALSFRS-R scores showed non-significant improvement post-treatment, 15/26 participants (57.7%) self-reported symptomatic improvement after treatment. Critically, cerebrospinal fluid biomarker analysis revealed significant reductions in neurofilament light chain (NfL; Δ530.29 pg/mL, *P* = 0.039) and glial fibrillary acidic protein (GFAP; Δ622.23 pg/mL, *P* = 0.038), indicating attenuation of neuroaxonal degeneration and astroglial activation. While ALSFRS-R scores showed no significant change (Δ-0.53, *P* = 0.384), prognostic modeling identified female sex (OR = 0.011, *P* = 0.008) and shorter disease duration (OR = 1.35/month, P = 0.005) as predictors of response. Three patients who underwent the second treatment were well tolerated without any adverse events.

**Conclusion:**

These findings indicate that Autologous SVF therapy might possess an acceptable safety profile for patients with ALS. The significant reduction in CSF NfL and GFAP levels provides objective evidence of their potential neuroprotective effects that modulates ALS-relevant neuroinflammation pathways. Female participants and those with shorter disease duration may derive greater benefits.

## 1 Introduction

Amyotrophic lateral sclerosis (ALS) is a progressive neurodegenerative disorder marked by the selective loss of motor neurons in both the brain and spinal cord. This degeneration affects upper motor cortical circuits and lower spinal neurons, disrupting signal transmission between the central nervous system and muscles, and ultimately impairing motor function. Clinically, ALS manifests as progressive muscle weakness, wasting, paralysis, and bulbar dysfunction involving difficulties in swallowing and breathing. These symptoms typically lead to respiratory failure and death within 3–5 years of onset (Hardiman et al., 2017; Kiernan et al., 2021; Hardiman et al., 2017; Kiernan et al., 2021). Currently, there is no effective treatment. Stem cell-based interventions have remained a prominent focus in both preclinical and clinical ALS research.

A central objective of stem cell-based trials in ALS is neuroprotection. Transplanted cells can exert immunomodulatory effects, secrete growth factors, and generate supportive cell types—such as astrocytes, oligodendrocytes, or interneurons—that help preserve compromised motor neurons (MNs) (Atassi et al., 2016; Berry et al., 2018) Neural stem cells (NSCs), whether derived from fetal tissue, induced pluripotent stem cells (iPSCs), embryonic sources, or other origins, often depend on viral transduction for expansion and differentiation. These cells are capable of releasing neurotrophic factors (Blurton-Jones et al., 2009) and display neuroplastic properties, positioning them as promising candidates for intracerebral transplantation (Ager et al., 2015).

Induced pluripotent stem cells (iPSCs) are a class of pluripotent cells generated by reprograming somatic cells. Although this technology has shown great potential in fields such as disease modeling and regenerative medicine (Wernig et al., 2008), its clinical application faces risks of genetic instability and oncogenic activation inherent to the reprogramming process itself (Takahashi and Yamanaka, 2006). Therefore, researchers propose stromal vascular fraction (SVF) from fat as an alternative cell therapy source.

SVF is a mixture of cytokines and cell clusters including hematopoietic cells, fibroblasts, endothelial cells, pericytes, adipose-derived stem cells (ADSCs), and MSCs (Mesenchymal Stem Cells, MSCs) isolated from adipose tissue through digestion and centrifugation (Bourin et al., 2013; Bora and Majumdar, 2017). It provides autologous therapy cells that bypass tissue compatibility barriers (Bora and Majumdar, 2017). Unlike MSCs, iPSCs, and NSCs (Neural Stem Cells, NSCs), SVF needs no culture amplification and can be prepared within hours after fat freeze-drying. It generates multiple cell types for regenerative applications (Koh et al., 2011), tissue repair, angiogenesis (Morris et al., 2015), and immune regulation (Riordan et al., 2009; Dong et al., 2013). SVF can treat various connective and supportive tissue diseases (Sahin and Türeci, 2018). CSF biomarkers like NfL (neurofilament light chain, NFL) and GFAP (glial fibrillary acidic protein, GFAP) indicate treatment response in neurodegenerative trials (Dreger et al., 2022). This prospective clinical study examined SVF’s effectiveness and safety in treating ALS patients. We report results from 26 participants followed for 2–3 months to demonstrate the safety of autologous fat harvesting and intravenous/intrathecal administration in ALS patients. No serious AEs were reported during the study period and 15 participants showed partial symptom improvement.

## 2 Materials and methods

### 2.1 Standard protocol approvals, registration, and patient consent

This project was a phase I, single-arm, prospective, open-label study using autologous adipose-derived SVF to treat ALS. It was registered with the Chinese Clinical Trial Registry (ChiCTR2400091754) and approved by the Ethics Review Committee of Suzhou Hospital Affiliated to Nanjing University School of Medicine (RB2024011). This trial ran from October 2024 to June 2025, with patient enrollment from October 2024 to April 2025. Written informed consent was obtained from all participants before enrollment.

### 2.2 Selection criteria

Inclusion criteria: ➀ Met the clinical and neurophysiological diagnostic criteria for the revised El Escorial MND; ➁ no structural damage to the brain and cervical cord, and no corresponding manifestation in neuroimaging; ➂ forced lung capacity > 30%; ➃a basic nutritional status and BMI of at least 18.5 kg/m^2^; ➄ ALSFRS-R score > 20 points; and ➅ the patient and/or their family members signed the informed consent form.

Exclusion criteria: ➀ Severe medullary damage with forced vital capacity < 30%; ➁ malnutrition (BMI < 18.5 kg/m^2^); ➂ undergone tracheotomy; ➃ a history of mental illness (such as schizophrenia); ➄ history of systemic diseases such as malignant tumors, cardiovascular diseases (including decompensated hypertensive heart disease, arrhythmia, ischemic heart disease), stroke, and coagulation dysfunction; ➅ cervical spondylosis or other diseases suggested by neuroimaging that may lead to clinical ALS symptoms; ➆ stimulation of the local area with metal; ➇ serious hearing and vision abnormalities; ➈ skull damage or loss; and ➉ contraindications for magnetic resonance imaging examination.

### 2.3 Study design

Research type: Prospective self-controlled study.

**FIGURE 1:**
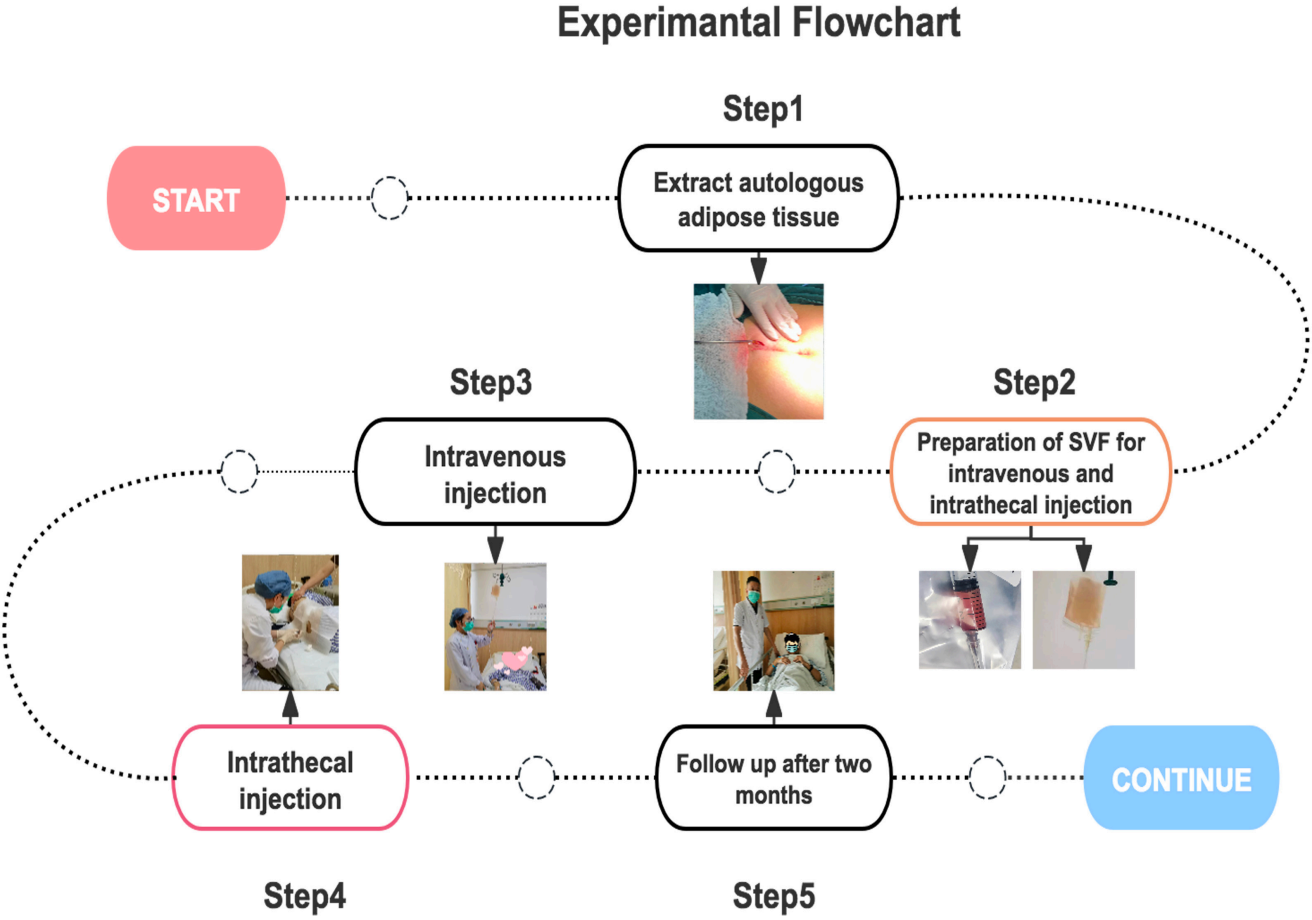
The experimental process of this study. Participants were screened according to the inclusion and queuing criteria. After anesthesia evaluation, an appropriate amount of adipose tissue was extracted, sent to the laboratory for SVF extraction, infusion, and injection. The infusion was slowly dripped through the vein and injected intrathecally. After treatment, the participants were observed for 24**–**48 h without adverse reactions and were discharged from the hospital. The participants were followed up several times over 45 days. After 45 days, we reviewed the blood biochemistry and disease assessment results. If the participants were willing, a second intrathecal injection was administered.

Intervention measures:

Intravenous infusion of autologous adipose-derived vascular stromal fraction (SVF) at a dose of 0.5 × 10^6^/kg (based on cell counting, described below), and simultaneous injection of SVF (20 × 10^6^ cells) into the lumbar sheath during each infusion (Figure 1).

Follow-up and Assessment:

The primary endpoint was safety, assessed by the nature, incidence, and severity of adverse events (AEs)-defined as any unfavorable medical occurrences (e.g., signs, symptoms, abnormal findings, or diseases) that emerged or worsened after baseline. Serious AEs included death, life-threatening events, hospitalization, disability, or congenital anomalies. For safety monitoring, blood cell counts and routine biochemical parameters were measured from samples collected at baseline and 45 days post-injection. Early AEs were those occurring within 2 weeks of administration, whereas late AEs manifested thereafter. AE data were gathered through participant interviews, medical records, clinical examinations, and spontaneous reports during follow-up visits. Secondary endpoints comprised changes in the ALS Functional Rating Scale–Revised (ALSFRS-R) score and manual muscle strength testing, with sensory and motor function assessments performed before treatment and at day 45.

### 2.4 SVF collection, preparation, and administration

Adipose tissue aspiration was performed as follows:

(1) Local or general anesthesia (according to patient needs), 0.5% lidocaine, adrenaline, and sodium bicarbonate were injected directly into the cutaneous nerve plexus using a syringe. Local anesthesia was injected into the adipose or deeper tissues. (2) A 50 mL syringe was connected to a lumbar puncture needle, which was inserted through the incision, and 20–40 mL of expansion fluid was injected in a fan shape at the liposuction site. (3) The fat was slowly aspirated after injecting the expansion fluid and 150–250 mL of fat was extracted. (4) After extracting the fat, it was placed into the fat transport solution and sealed with a sealing film to ensure sterility.

Preparation of vascular matrix components derived from autologous fat:

(1) Cleaning solution preparation: A 500 mL DPBS + 10 mL dual antibody solution was prepared.
(2) Cleaning adipose tissue: Every 30 mL of fat required 50 mL of cleaning solution. After flipping up and down, it was allowed to stand for 1–2 min. After layering, the cleaning solution was absorbed and the process was repeated 5 times.
(3) Preparation of collagenase: DMEM was used to prepare a 1 mg/mL solution of collagenase, which was filtered through a 0.22 μm filter, and set aside for later use.
(4) The cleaned adipose tissue was transferred to the collagenase and the tube opening was sealed with a sealing membrane.
(5) Digestion: The tube was placed in a constant temperature shaker and digest at 37 °C and 200 r/min for 45 min.
(6) Centrifuge: After digestion, the adipose tissue was centrifuged at 20 °C and 1100 r/min for 8 min.
(7) After centrifugation, the supernatant was removed, and the pellet was retained.
(8) Collagenase neutralization: DMEM was added to 30 mL of cell pellet and to gently resuspend it.
(9) The cell suspension was filtered through a 100 um filter.
(10) Centrifugation: The filtered cell suspension was centrifuged at 20 °C and 1,100 r/min for 5 min.
(11) The supernatant was removed, and the pellet was retained.
(12) Physiological saline was added to 40 mL of the cell pellet for resuspension.
(13) Centrifugation: At 20 °C and 1,100 r/min for 5 min.
(14) The supernatant was removed, and the pellet was retained.
(15) Steps 16–18 were repeated thrice.
(16) The pellet was resuspended with 5 mL of physiological saline, then supplemented to 8.5 mL with physiological saline.
(17) Cell suspension was filtered through a 40 um filter.
(18) Filtered cell suspension was collected into a 10 mL syringe for later use.

Quality testing of vascular matrix components derived from autologous fat sources

Referring to General Rule 0102: “Injection of the Pharmacopeia of the People’s Republic of China,” Volume 4 (2020 edition), we conducted the following tests on the SVF injection:

➀ Presence of foreign objects
➁ Asepsis (microscopy/gram staining direct inoculation culture method)
➂ Endotoxin
➃ Cell volume.

### 2.5 Assays and samples

All samples were derived from the serum and CSF collected from the participants before and approximately 45 days after SVF treatment. Participants with jaundice, lipemia, or hemolysis were excluded. EDTAK2 blood collection tubes (Sanli Medical Technology Development Co., Ltd., Liuyang, China) were used. Within 2 h of sampling, plasma was separated from blood cells by centrifugation at 1,200 × g for 10–15 min at 25°C (ultracentrifugation is recommended for hyperlipidemic or chylous blood samples). Unless otherwise stated, all samples were frozen at –80°C and equilibrated to ambient temperature before testing.

According to the manufacturer’s instructions, the Chemclin LiCA^®^ p-tau217, p-tau 181, IL-6, NfL, and GFAP assay kits are used to quantitatively measure p-tau217, p-tau 181, IL-6, NfL, and GFAP on the Chemclin 800 analyzer (Chemclin Diagnostics, Beinjing, China). Cleaning, centrifugation, and dilution were not required before measuring the samples. To detect p-tau217, p-tau181, IL-6, NfL, and GFAP, 40, 40, 30, 30, and 30 μL of serum or CSF samples are required for measurement, respectively.

Plasma samples were incubated with 200 nm diameter ChemiBeads (chemical microspheres) coated with detection antibodies (biotinylated capture antibodies); if target antigens are present, a sandwich immune complex is formed. Then, SensiBeads (photosensitive microspheres) bound to streptavidin are added and bind to the biotin on the formed immune complex. Once excited by a 680 nm laser, the SensiBeads generate singlet oxygen molecules that can diffuse into ChemiBeads. The chemical energy transferred to ChemiBeads initiates a chemiluminescence reaction. If the sample does not contain the analyte, an immune complex is not formed. The large gap (> 200 nm) between the free beads hinders the transfer of chemical energy and prevents the generation of light signals. The entire assay process is homogeneous and does not require washing steps.

### 2.6 Statistics

Statistical analysis was performed using SPSS software (version 26.0). Normality of continuous variables was assessed via the Kolmogorov–Smirnov test. Data with a normal distribution are presented as mean ± standard deviation (SD); inter-group comparisons were made using independent-sample *t*-tests or paired *t*-tests (e.g., for pre-and post-treatment laboratory values). Non-normally distributed data are expressed as median and interquartile range [M (IQR)], with group comparisons conducted using the Mann–Whitney *U*-test. Categorical variables are summarized as frequencies and percentages (n, %), and differences between groups were evaluated using the chi-square test or Fisher’s exact test (when expected cell counts were < 5).

For the results of ordered multiclass efficacy (ineffective/stable/effective), ordered logistic regression (PLUM process) was used to analyze the predictive factors, and the hypothesis of proportional advantage was validated through parallel line tests. For the binary efficacy results (effective vs. ineffective + stable), a binary logistic regression analysis was used to predict the factors, and odds ratios (OR) and 95% CI were calculated. When the sample size was insufficient, and the multivariate model failed, univariate screening was performed using a score test. The goodness of fit of the model was evaluated using the following indicators: pseudo *R*^2^ (Cox and Snell/Nagelkerke) and goodness of fit test (Pearson/Deviance); for non-normally distributed CSF biomarkers (Shapiro-Wilk *P* < 0.05), Wilcoxon signed-rank tests were used; others employed paired *t*-tests. According to the Information Criteria (AIC/BIC), all tests were bilateral, and *P* < 0.05 was considered statistically significant.

## 3 Results

### 3.1 Baseline characteristics

The total duration of this study was from October 2024 to June 2025. Twenty six participants were recruited for the first SVF treatment. Their condition was evaluated at the 45-day follow-up. Of the 26 participants, 69.2% (*n* = 18) were male and 30.8% (*n* = 8) were female, all of whom were Asian. The average age was 52.42 years (52.42 ± 10.30 years), with an average height of 166.54 cm (166.54 ± 8.52 cm), an average weight of 67.07 kg (67.07 ± 12.49 kg), a BMI index of 24.10 ± 3.25, an average disease duration of 29.46 ± 20.33 months (10–92 months), and a pre-treatment ALSFRS-R score of 26.42 ± 5.88 (Table 1).

**TABLE 1:** Baseline characteristics of selected participants.

Variable	Minimum	Maximum	Mean	Std. Deviation
Age	29	69	52.42	10.296
Height	150	185	166.54	8.515
Weight (kg)	42.0	112.0	67.073	12.4908
BMI	18.67	32.72	24.0988	3.25415
Duration(m)	10	92	29.46	20.332
ALSFRS-R	15	35	26.42	5.880

### 3.2 Safety and adverse events

Among the 26 participants, 5 AEs were reported: 2 cases of subcutaneous bleeding, 1 case of low-grade fever (subsiding after treatment), 1 case of mild headache, and 1 case of mild headache and back pain, all improving after treatment. One patient experienced significant waist and buttock pain on day 2 of the second SVF intrathecal injection, which improved after oral painkillers. No serious AEs occurred during the study. An exploratory second dose of SVF was administered intrathecally to three patients 45 days later, and was well tolerated without any adverse events. Three patients received an exploratory second intrathecal injection of SVF 45 days later without any adverse reactions (in compliance with ethical requirements, without undergoing NFL, GFAP, or other tests).

### 3.3 Efficacy assessment

Among the 26 participants, 7 discontinued treatment. Baseline ALSFRS-R scores (*n* = 19) were 26.84 ± 5.72, and after approximately 45 days of treatment, the ALSFRS-R score was 27.37 ± 5.91. Although there was a slight improvement, the difference was not significant (t = –0.893, *n* = 19, *P* = 0.384). Although SVF treatment did not show a significant improvement in the overall ALSFRS-R clinical score, we conducted a detailed follow-up of the participants (Supplementary Information) and classified their subjective feelings. We defined symptom improvements as effective; no improvement but no progression as stable; and symptoms progressing or perceived as ineffective. After first SVF treatment, 15 participants (57.7%) were effective, 4 stable (15.4%), and 7 ineffective (26.9%).

### 3.4 Factors influencing patient prognosis

The patient’s first subjective evaluation post-treatment was the dependent variable, with age, sex, BMI, disease duration, and first ALSFRS-R score as covariates in the ordered regression analysis. Results showed a likelihood ratio of 23.596, df = 5, *p* < 0.001, indicating statistical significance of the overall model. The parallel line test (chi-square = 2.128, *p* = 0.831) satisfied parallelism for continued analysis. The pseudo-*R*^2^ value (Nagelkerke = 0.699) showed the model explained 69.9% of variation with good fit. The Pearson test showed chi-square of 28.928 and *P*-value of 0.97, indicating good model fit. The deviance test result has a chi-square of 26.251 and a *P*-value of 0.988 > 0.05, indicating a good fit of the model. Males have poorer efficacy [OR < 1, 95% confidence intervals (CI) (0.000, 0.312)] while females are 98.9 times more likely to show better efficacy (1/0.011, *P* = 0.008) [95% CI (0.000, 0.312)]. Disease duration significantly affects outcomes: each month increase raises the probability of transitioning to a worse efficacy level (effective > stable > ineffective) by 35.3% (1/1.353, *P* = 0.005) [95% CI (1.095, 1.672)]. Age, BMI, and initial ALSFRS-R score showed no significant effects on treatment efficacy (*P* = 0.300, 0.225, and 0.127). The thresholds for efficacy levels were: ineffective vs. stable/effective threshold 1 = 2.047 (*p* = 0.684); stable vs. effective: threshold 2 = 3.842 (*p* = 0.447), indicating unclear classification boundaries possibly due to sample size or data distribution (Table 2).

**TABLE 2:** Analysis of factors influencing patient prognosis.

Variable	B	Std. error	*P*-value	Exp(B)	95% CI for Exp(B)	
					Lower	Upper
Threshold (1st self-evaluation = 1)	2.047	5.029	0.684	7.746	0.000	147742.889
Threshold (1st self-evaluation = 2)	3.842	5.049	0.447	46.635	0.002	925167.161
Age	0.057	0.055	0.300	1.059	0.951	1.179
Gender	–4.477	1.690	0.008	0.011	0.000	0.312
BMI	–0.230	0.190	0.225	0.795	0.548	1.152
Duration (m)	0.302	0.108	0.005	1.353	1.095	1.672
Pre-ALSFRS-R	0.208	0.136	0.127	1.231	0.943	1.609

### 3.5 Changes in relevant biomarkers in cerebrospinal fluid

**FIGURE 2:**
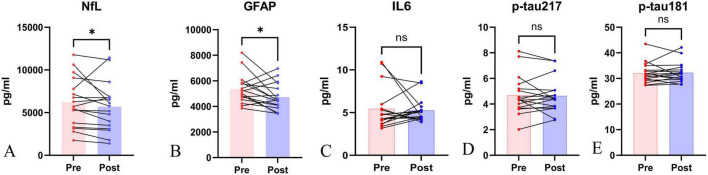
CSF **biomarker changes a**fter SVF **therapy.** There were statistically significant changes in NfL and GFAP levels in CSF after SVF treatment (*Z* = -2.059, *P* = 0.039; T = 2.255, *P* = 0.038), whereas no statistically significant changes were observed in IL-6, p-tau-217, and p-tau-181 levels. Bar graphs showing pre- vs. post-treatment levels of NfL and GFAP (*P < 0.05). Error bars = SD. Forest plot of mean differences (95% CI). All the participants provided written informed consent.

To observe changes in CSF markers after SVF treatment, we used nano-homogeneous chemiluminescence technology to measure p-tau-217, p-tau-181, IL-6, NfL, and GFAP levels before and 45 days after treatment. The Shapiro-Wilk test assessed whether biomarker differences followed normal distribution. IL-6 and NfL levels deviated significantly from normal distribution (*P* = 0.008 and *P* = 0.034). For GFAP, p-tau-217, and p-tau-181, we used the Wilcoxon signed-rank test and paired sample *t*-test to determine distribution. Results showed significant changes in NfL and GFAP levels after SVF treatment (*Z* = -2.059, *P* = 0.039; *T* = 2.255, *P* = 0.038), while IL-6, p-tau-217, and p-tau-181 levels showed no significant changes (Table 3 and Figure 2).

**TABLE 3:** Changes in relevant biomarkers in cerebrospinal fluid.

Biomarker	n	Pre-SVF	Post-SVF	Z or T	P
NfL (pg/mL)	17	6223.12 ± 2876.58	5692.83 ± 2882.67	-2.059	0.039
GFAP (pg/mL)	17	5352.39 ± 1133.45	4730.16 ± 1048.22	2.255	0.038
IL-6 (pg/mL)	17	5.45 ± 2.44	5.27 ± 1.37	0.474	0.636
p-tau-217 (pg/mL)	17	4.70 ± 1.53	4.65 ± 1.35	0.266	0.794
p-tau-181 (pg/mL)	17	32.12 ± 3.99	32.28 ± 3.92	0.226	0.824

## 4 Discussion

ALS a progressive neurodegenerative disease characterized by motor neuron death in the brain and spinal cord. It is a destructive disease with limited treatment options. Clinical research on ALS has increased, providing treatment prospects. Although gene-targeting methods show potential to treat participants with specific mutations, this therapy remains limited (Faller et al., 2025), with few stem cell clinical studies. Treatment of motor neurons’ pathological basis in ALS has considerable application prospects. Stem cell applications offer hope when conventional treatments cannot prevent degenerative changes (Petrov et al., 2017; Berry et al., 2018). Cell therapy benefits include regeneration through paracrine signaling and growth factor secretion (Fan et al., 2023). Major challenges in using MSCs, NSCs, or iPSCs include generating sufficient live cells, enhancing metabolic activity, sustaining cell communication, and mimicking relevant cell systems (Fan et al., 2023; Izrael et al., 2025). While allogeneic cell products reduce harvest variability and donor incompatibility, they face challenges in donor health, immune rejection, and cost (Lo and Parham, 2010; Seminatore et al., 2010; Izrael et al., 2025).

SVF is an active substance in body fat, composed of cells from various sources, including ADSC and other mature and precursor cells (Jung et al., 2016). It provides autologous therapy for diseases where effector cells bypass the main histocompatibility barrier (Bora and Majumdar, 2017). Adipose tissue, distributed under adult skin, can be collected repeatedly and serves as an adult stem cell bank. Adipose-derived mesenchymal stem cells form stromal vascular components for SVC treatment, having immunomodulatory functions and low immunogenicity. Xenotransplantation can overcome immune rejection (Le Blanc et al., 2003; Tse et al., 2003). Stem cells secrete soluble factors and vesicles to regulate immune cells (Griffin et al., 2013; Lohan et al., 2014), inhibit cell apoptosis, reduce inflammation and fibrosis, and promote angiogenesis (Galderisi and Giordano, 2014). SVF requires no culturing or amplification and can be prepared within hours. It produces various cell types and alleviates disease progression through immune regulation, providing neuroprotection for neurodegenerative diseases by targeting multiple degenerative mechanisms (Sulzer et al., 2017).

This study conducted a trial on ALS treatment with vascular stromal fraction (SVF). Twenty-six participants received SVF intravenous or intrathecal injection therapy. After treatment, most participants showed slight, but not significant, improvement in ALSFRS-R score. We classified participants’ subjective perceptions of the effect. After the first SVF treatment, the majority of participants perceived the treatment as effective. Currently, only a few clinical studies have been conducted on stem cell therapy for ALS. Gotkine et al. (2023) administered intrathecal injection of human astrocytes (AstroRx) to five ALS participants. Both low-dose and high-dose AstroRx^®^ administration was safe and well tolerated, with no related adverse events. Beneficial clinical effects were observed in the first 3 months post-injection. Barczewska et al. (2020) studied umbilical cord stem cells in 67 ALS participants, observing three ALSFRS-R response types: decreased progression (31.3%), unchanged progression (49.3%), and increased progression (19.4%). The risk-benefit ratio was favorable, with no serious adverse reactions. Female sex and first-time treatment showed better responses. Our study confirmed women had a considerably higher treatment efficacy than men, and shorter disease duration improved efficacy, with each additional month increasing the probability of worse outcomes. Age, BMI, and initial ALSFRS-R score showed no significant effect on treatment efficacy.

SVF may mitigate disease progression by modulating immune activity (Bowles et al., 2017). SVF can be used for various connective and supporting tissue diseases (Ma et al., 2013). Studies show it can alleviate disease progression through immune regulation, providing neuroprotection. While stem cell regeneration shows promise, treatment mainly improves symptoms rather than preventing damage (Allgaier and Allgaier, 2014; Dykstra et al., 2017). Studying SVF therapeutic strategies in neurodegenerative diseases is crucial, targeting multiple pathological mechanisms through immune process control and lymphocyte stabilization (Mehta et al., 2017; Faller et al., 2025). SVF provides “cell communication” that regulates tissue matrix per specific needs. Our results showed inflammatory and immune levels increased after SVF treatment. Evaluating SVF treatment requires objective indicators like PET tracking of alpha synuclein protein, NfL, and molecular markers to determine disease changes, beyond clinical signs and ALSFRS-R scores.

We observed reductions in CSF NfL and GFAP levels, providing evidence of SVF therapy’s neuroprotective efficacy in ALS (Dreger et al., 2022). NfL, a biomarker of neuroaxonal damage in ALS (Dreger et al., 2022; Verde et al., 2025), declined significantly 45 days post-treatment, suggesting SVF mitigates motor neuron degeneration. This aligns with studies showing NfL dynamics correlate with therapeutic response (Dreger et al., 2022; Wilson et al., 2022). Decreased GFAP indicates reduced astrogliosis, a key driver of neuroinflammation in ALS (Gendron and Petrucelli, 2023). Unaltered IL-6 levels suggest SVF’s effects involve direct modulation of glial activation rather than systemic inflammation suppression (Jericó et al., 2023). Stable p-tau-181/217 levels, given tau’s minimal role in ALS (Van Daele et al., 2023; Dellar et al., 2025), underscore SVF’s disease-specific neuroprotection. With no effective ALS treatments, stem cell applications offer hope through regenerative microenvironment initiation (De Gioia et al., 2020). Major challenges in using MSCs, NSCs, and iPSCs include generating sufficient live cells and sustaining cell communication (Mehta et al., 2017; Ramakrishnan and Boyd, 2018). While allogeneic cell products reduce variability, they face issues of immune rejection and cost (Li et al., 2008; Lo and Parham, 2010). Our research indicates that SVF from autologous fat might serve as a valuable alternative therapy for ALS treatment.

The concordance between reduced CSF NfL/GFAP ratio and clinical stabilization suggests SVF may disrupt neuroinflammation-neurodegeneration. Future studies correlating biomarker changes with functional outcomes are needed to validate their utility as SVF therapy endpoints. In our initial analysis, In our preliminary analysis, exploratory analysis of the interaction between gender and disease duration did not show statistical significance (*P* = 0.42). Due to the small sample size, we will further explore it in the later phase 2 clinical study.

## 5 Conclusion

This phase I trial provides initial clinical evidence suggesting that autologous adipose-derived SVF therapy may be safe and potentially effective for ALS. The treatment demonstrated an excellent safety profile with no serious adverse events. Most importantly, the observed significant reductions in CSF neurofilament light chain (NfL) and glial fibrillary acidic protein (GFAP) levels offer objective, biomarker-based evidence of a neuroprotective effect, attenuating neuroaxonal injury and astroglial activation. The identification of female sex and shorter disease duration as strong predictors of subjective therapeutic response highlights a potential path toward personalized treatment strategies. While these findings are promising and justify further investigation, they are derived from an open-label, single-arm study with a limited sample size. Larger, randomized, placebo-controlled trials are essential to confirm efficacy, define the optimal treatment protocol, and elucidate the underlying mechanisms of action. This study proposes that autologous SVF might represent a viable and innovative candidate for future cell-based therapeutic development in ALS.

## Data Availability

The original contributions presented in this study are included in the article/Supplementary material, further inquiries can be directed to the corresponding authors.
