# Supplementary material for: Autologous SVF therapy modulates neuroinflammation in ALS: phase I trial demonstrating safety and CSF biomarker dynamics
Source: Front Aging Neurosci. 2026 Mar 31;18:1784115. doi: 10.3389/fnagi.2026.1784115 (PMC13076307; doi:10.3389/fnagi.2026.1784115)
Supplement: Supplementary file 1 [file Table_1.docx]

Supplemental Information: multiple follow-up information

| Patient number | Multiple follow-up information | Is there any adverse reaction for the first time | Is there any adverse reaction for the second time |
| --- | --- | --- | --- |
| ALS-1 | 2025-03-04 ：after the first injection, there was neck pain on the same day, which relieved after 2 days and the neck changed from stiff to able to tilt back.There is no change after the second injection. 2025-04-15 :After 2 months of treatment , the improved neck strength returned to before treatment. The grip strength of the right hand slightly decreased, and the muscle strength of the upper and lower limbs and upper arms remained unchanged. The overall muscle strength began to decline significantly one month before treatment, but the overall decline was not significant after six months of treatment.Cost issue, do not consider continuing treatment. |  |  |
| ALS-2 | invalid |  |  |
| ALS-3 | 2025-03-04： the family reported that the condition remained stable and did not progress after treatment, but progressed slowly before treatment. 2025-03-04：the condition remained stable with no progress. Other treatments are being attempted and follow-up treatment is not being considered. 2025-03-04：the condition remained stable with no progress, and other treatment options are still being attempted (unknown).Follow up on WeChat in the later stage |  |  |
| ALS-4 | 2025-03-06：Within 3 days after the surgery, patient felt that my whole body was relaxed, and then returned to the preoperative state again. Ccondition stabilized after the surgery and did not progress or worsen. 2025-04-15: the condition is slowly worsening and treatment will not continue due to distance reasons. |  |  |
| ALS-5 | invalid |  |  |
| ALS-6 | invalid | Headache and lower back pain |  |
| ALS-7 | 2025-03-04: hand grip strength improved, grip stability improved, muscle tone improved, arch pain improved, and speaking time prolongedThe clarity has increased, but there has been no improvement in other aspects, and there has been no overall progress. Did not answer the phone on March 21, 2025 |  |  |
| ALS-8 | 2025-03-04: There was no change during the follow-up on March 4, 2025.  2025-03-18: the video showed an improvement in the left hand lifting ability, no change in the strength of the right upper limb, and a slight decrease in the strength of the right thumb level index finger. 2025-4-24: there was a slight improvement in left upper limb weakness, and the flexibility of the right finger decreased slowly compared to before surgery. |  |  |
| ALS-9 | 2025-03-04: coughing slightly decreased. 2025-04-15: Improvement in coughing, with no significant change in overall muscle strength. |  |  |
| ALS-10 | 2025/3/4; both hands showed slight improvement in flexibility, pronunciation, and neck and waist muscle strength. 2025/3/10: Coughing has improved in the past two weeks , and the neck and lumbar spine have recovered as before (improved 3 days after surgery), with no other changes. 2025-3-21: there was a slight decrease in neck muscle strength, slow speech, and no change in coughing. 2025-4-1: Improvement in swallowing, with a conscious thinning of the left leg and no change in muscle strength. |  |  |
| ALS-11 | 2025-02-24: there was a headache with low intracranial pressure and lower back pain.  2025-03-04: lower back pain was relieved and neck strength slightly improved. 2025-04-15: I still feel pain at the lumbar puncture point, and my overall muscle strength and vocal strength are slowly decreasing | Headache and lower back pain |  |
| ALS-12 | 2025-03-04: there was an improvement in pronunciation intensity, an extension of speaking time, a reduction in flesh jumping, and no progress in other stable aspects. 2025-4-15 : Starting from the third day after the first course of treatment, there will be an improvement in pronunciation intensity and clarity, as well as an improvement in coughing when drinking water, which will continue to be maintained; In the past month, there has been a slight decrease in the strength of the proximal end of the right upper limb, improvement in the grip strength of the right hand, and a stronger grip on the phone compared to before. | */* | On the second day, there was significant pain in the waist and buttocks, which persisted overnight. Oral painkillers improved on the third day |
| ALS-13 | 2025-03-04: hand strength improved and toes became more conscious and slightly movable.Language, no changes in the upper and lower limbs, no further progression or aggravation. 2025-04-15: the grip strength of both hands began to decrease 10 days ago, which is slightly worse than before treatment.After toe treatment, the back extension ability improved and remained unchanged,  with no changes in language or upper arms or thighs. |  |  |
| ALS-14 | 2025-03-04 Abdominal wound dull pain.The strength and flexibility of hands and feet, as well as the strength of the neck, have slightly improved. 2025-04-02: On the evening, there was a slight improvement in neck stiffness after two surgeries. 2025-04-15: No changes. | / |  |
| ALS-15 | 2025-03-05: the left hand can hold a spoon to deliver food to the mouth (before surgery, it can only bend to a distance of 10 centimeters in front of the mouth), and the lower abdominal wound is slightly painful. 2025-04-15: Recently, there has been swelling in both the right and left hands, which persists for 2-3 days and resolves on its own. After 20 days of improvement in left hand strength, it began to decline to the preoperative state, and the overall muscle strength was similar to before surgery. |  |  |
| ALS-16 | 2025-04-15: Preoperative dizziness completely relieved after surgery, with no significant changes in speech level muscle strength or progression trend. |  |  |
| ALS-17 | 2025-04-15: there were no discomfort symptoms after surgery. The speaking and lower limb walking strength slowly worsened, and the rate of conscious aggravation seemed to be delayed compared to before surgery.There is no change in hand strength. |  |  |
| ALS-18 | 2025-04-15: On the second day after surgery, there was a transient fever of 37.6 degrees Celsius, which subsided after taking antipyretic medication orally. After 10 days of surgery, there was improvement in the strength of the right upper limb and the inability to lift it before surgery. With postoperative assistance, it was possible to lift and touch the head, and it continued for 10 days before returning to the preoperative state. Postoperative shoulder and back pain has been relieved, with no changes in muscle strength or speech, and no worsening. | low heat |  |
| ALS-19 | 2025-04-15: speech and limb muscle strength continued to worsen, and recent walking instability occurred.Abdominal incision with dull pain. | Subcutaneous bleeding |  |
| ALS-20 | 2025-04-15: the muscle strength of the limbs at the speech level continued to slowly decline, resulting in choking on water, swallowing disorders, and unstable walking. Early postoperative lower back pain and abdominal pain, gradually improving. |  |  |
| ALS-21 | 2025-04-15: WeChat Video , Language and hand strength are still slowly decreasing without any discomfort symptoms. |  |  |
| ALS-22 | 2025-04-15: the language and left hand strength slowly decreased, and the lifting strength of the left leg improved (it is currently possible that the wheelchair cannot lift the pedal on its own before surgery). Slight discomfort under the skin during abdominal incision. |  |  |
| ALS-23 | 2025-04-15: I experienced pain while pressing on my lumbar puncture site in recent days.A few days after surgery, the strength of both lower limbs improved, the overall condition improved, and the flexibility of using the mouse with both hands improved.There is no change in language, and overall there is no progress or aggravation. |  |  |
| ALS-24 | 2025-04-15: Observation of son: No discomfort, overall no progress, overall mental state compared to before surgery. |  |  |
| ALS-25 | 2025-04-15: the abdominal congestion has been absorbed and there is currently no discomfort, overall no improvement, and no progress. | Subcutaneous bleeding |  |
| ALS-26 | 2025-04-15: there was an improvement in lower limb strength when standing and walking on both legs. Preoperative hand assistance enabled the patient to move from sitting to standing on their own, but currently requires assistance from family members to stand. |  |  |
